# 480. Alternate Diagnoses in Children Evaluated for Multisystem Inflammatory Syndrome in Children (MIS-C)

**DOI:** 10.1093/ofid/ofab466.679

**Published:** 2021-12-04

**Authors:** Kelli Kaneta, Sindhu Mohandas, Jackie Szmuszkovicz, Sarah White, Susan Wu

**Affiliations:** 1 Children’s Hospital Los Angeles, Los Angeles, California; 2 Children Hospital Los Angeles, los angeles, CA

## Abstract

**Background:**

SARS-CoV-2 infection is typically a mild illness in children. Multisystem inflammatory syndrome in children (MIS-C) is a rare, post-infectious, hyperinflammatory condition associated with SARS-CoV-2 infection. The presentation of MIS-C is nonspecific and diagnostic criteria is broad. The Centers for Disease Control (CDC) defines MIS-C as a hospitalized patient < 21 years presenting with fever, laboratory evidence of inflammation, no alternative plausible diagnosis, and with positive exposure history or testing for current or recent SARS-CoV-2 infection. Since there is no single diagnostic test for MIS-C, there are other disease processes that can mimic its presentation and delay prompt diagnosis and management.

**Methods:**

Between March 2020 and February 2021, we reviewed 282 charts of patients admitted for evaluation of MIS-C at our institution.

**Results:**

101 were found to have MIS-C, 45 found to have Kawasaki Disease (KD), and 129 were ruled out. Of the ruled-out group, the most common final diagnoses were viral infection, urinary tract infection, and acute SARS-CoV-2 infection. Other diagnoses included rickettsial infections, pneumonia, rheumatologic conditions, and bloodstream infection. Rhinovirus/enterovirus, adenovirus, Epstein-Barr virus (EBV), and Herpes Simplex Virus (HSV) were the most common viruses other than SARS-CoV-2 identified.

**Conclusion:**

These findings highlight the importance of maintaining a broad differential when evaluating a patient for MIS-C, especially as community seroprevalence rises, making antibody presence less predictive of MIS-C.

**Disclosures:**

**Susan Wu, MD**, **Eli Lilly** (Shareholder)

